# Co-speciation in bedbug *Wolbachia* parallel the pattern in nematode hosts

**DOI:** 10.1038/s41598-018-25545-y

**Published:** 2018-06-11

**Authors:** Ondřej Balvín, Steffen Roth, Benoit Talbot, Klaus Reinhardt

**Affiliations:** 1 Department of Ecology, Faculty of Environmental Sciences, Czech University of Life Sciences Prague, Kamýcká 129, CZ-165 21 Praha 6, Czech Republic; 2 The Natural History Collections, University Museum of Bergen, P.O. Box 7800, N-5020 Bergen, Norway; 3 Department of Biology, University of Western Ontario, 1151 Richmond Street, London N6A 3K7 Ontario, Canada; 4 Technische Universität Dresden, Department of Biology, Applied Zoology, D-01069 Dresden, Germany

## Abstract

*Wolbachia* bacteria, vertically transmitted intracellular endosymbionts, are associated with two major host taxa in which they show strikingly different symbiotic modes. In some taxa of filarial nematodes, where *Wolbachia* are strictly obligately beneficial to the host, they show complete within- and among-species prevalence as well as co-phylogeny with their hosts. In arthropods, *Wolbachia* usually are parasitic; if beneficial effects occurs, they can be facultative or obligate, related to host reproduction. In arthropods, the prevalence of *Wolbachia* varies within and among taxa, and no co-speciation events are known. However, one arthropod species, the common bedbug *Cimex lectularius* was recently found to be dependent on the provision of biotin and riboflavin by *Wolbachia*, representing a unique case of *Wolbachia* providing nutritional and obligate benefits to an arthropod host, perhaps even in a mutualistic manner. Using the presence of presumably functional biotin gene copies, our study demonstrates that the obligate relationship is maintained at least in 10 out of 15 species of the genera *Cimex* and *Paracimex*. The remaining five species harboured *Wolbachia* as well, demonstrating the first known case of 100% prevalence of *Wolbachia* among higher arthropod taxa. Moreover, we show the predicted co-cladogenesis between *Wolbachia* and their bedbug hosts, also as the first described case of *Wolbachia* co-speciation in arthropods.

## Introduction

Interspecific interactions that provide fitness benefits to all partners involved characterizes a large part of the world’s biodiversity^1,2^. One of its prime examples are symbiotic bacteria that are mutualistically connected with their metazoan hosts^3^. However, the degree of association and type of mutualism may vary among bacteria-metazoan species pairs and depend on environmental and community contexts^2^. Typical primary symbioses and true mutualisms are characterised by individual bacterial taxa that provide benefits to their host. Benefits to bacteria are rarely measured^2^ but may be implied if symbionts are restricted to specialised host cells and tissues, and are exclusively transmitted vertically. Secondary symbioses are characterized by more generic, not necessarily host-specific benefits and consequently, bacteria vary in prevalence across cells, tissues and populations of the host. Their effects on a given host may range from pathogenic to mutualistic, and they can be transmitted either vertically, horizontally, or both^3^.

The mode of symbiont transmission may profoundly affect patterns of phylogenetic co-variation between the symbionts and their hosts. In cases of vertical transmission, symbionts benefit from increased host fecundity, and therefore, selection typically favours mutualistic symbioses^4^. In contrast, horizontally transmitted symbionts show less dependence on their hosts and even often develop into parasites. Mutually beneficial symbiotic relationships are more likely to lead to congruent lineage divergence in populations of the two partner species than are non-beneficial relationships^4,5^, in which hosts usually show resistance to parasitic symbionts^6^. Over evolutionary time scales, the higher co-divergence in beneficial than non-beneficial interacting lineages results in co-speciation, displayed by congruent phylogenies of symbionts and hosts on higher taxonomic levels^6^. The depth and completeness of the co-cladogenesis pattern has, therefore, been used as a measure of the nature of a particular symbiotic relationship^6^. In contrast to mutualist relationships, only few cases of co-cladogenesis have been described from parasitic relationships, mainly from ectoparasites (e.g. Hafner’s classical gopher study^7^), but almost none have been reported from single cell parasites living inside host body or cells^6^.

*Wolbachia* bacteria are a prominent example of mainly vertically transmitted intracellular endosymbionts. The *Wolbachia* diversity has been classified into supergroups^8^, of which up to 17 has been described up to date^9^. Importantly, across their two major host groups, arthropods and filarial nematodes, *Wolbachia* vary in whether and how much they benefit their host^10^. In filarial nematodes, *Wolbachia* are exclusively mutualistic^11^ and are characterised as primary symbionts. For example, in different hosts, they are involved in the heme synthesis pathway^12^ or in ATP provision^13^, or are crucial for the host’s iron metabolism^14^ and riboflavin provision^15^. Nematode-associated *Wolbachia* are usually found in all individuals of a species^16,17^ and in all species within larger clades^9^. In these associations, *Wolbachia* seems to be exclusively vertically transmitted, which is regarded as a sign of host-provided benefits. As predicted, the mutualistic nature between *Wolbachia* and nematodes usually results in a tight co-speciation^9,14,18^.

By contrast, in arthropods, *Wolbachia* are typically parasitic but can provide benefits to hosts^4^. *Wolbachia* incidence is reported to be either around 66%^19^, or 40%^20^ among arthropod species. Infections often cause host phenotypes with distorted reproduction (reproductive phenotypes, or RPs)^10,21^. The most common RP is the induction of cytoplasmic incompatibility (CI), where infected females are only able to produce offspring with infected males, and in some cases only with males infected with the same *Wolbachia* strain^10^. Other RPs involve the killing or feminization of genetic males^22^ or the induction of parthenogenesis^23^. These RPs directly or indirectly increase the proportion of infected females and *Wolbachia* are, therefore, able to thrive without providing a fitness benefit to the host, despite vertical transmission and dependence on host fecundity.

Theory predicting that fitness benefits to the host may increase symbiont propagation has been confirmed empirically for *Wolbachia*, even while maintaining the parasitic mechanisms^4^. For example, the hosts of CI-inducing *Wolbachia* often display increased fecundity. This leads to a net benefit to the host, if *Wolbachia* prevail in the population. Following this path, in some arthropods *Wolbachia* have become an irreplaceable element of the host´s reproduction. For example, *Wolbachia* controls the apoptosis during oogenesis in *Drosophila*^24^ and in the wasp *Asobara tabida*^25^, and serves as a sex-determining factor through chromosome formation in the bean borer moth *Ostrinia scapularis*^26^. However, despite these sometimes strict dependencies, and unlike in nematodes, there is no evidence for co-cladogenesis of *Wolbachia* and arthropods^20^ with a single exception^27^, which is however ambiguous because horizontal transfer of *Wolbachia* could not have been ruled out. Indeed, horizontal transmission of *Wolbachia* among arthropods is frequent^28–30^.

*Wolbachia* in the common bedbug, *Cimex lectularius*, are a notable exception to both rules - because they do not cause RP and they are primary symbionts. Hosokawa *et al*.^31^ demonstrated that *Wolbachia* provide biotin and riboflavin, which are essential for bedbug development. In line with an obligatory nature of the relationship, there is a 100% prevalence of *Wolbachia* in *Cimex lectularius* populations^32^. The gene pathway responsible for the synthesis and provision of biotin to the bedbug has been horizontally transmitted from a co-infecting *Cardinium* or *Rickettsia*^33^. The loci in the closely related *Cimex japonicus* host contained two deletions relative to *Cimex lectularius*. The authors, Nikoh *et al*.^33^, therefore concluded that the biotin production in *C*. *japonicus* is dysfunctional, but suggested that its origin lies in a common ancestor of the two *Cimex* species. In contrast to the laterally acquired biotin genes, the pathway for riboflavin provided to the common bedbug is fully maintained and homologous across *Wolbachia* in all hosts studied so far^34^. However, the common bedbug is the only arthropod known to be provisioned by riboflavin produced by *Wolbachia*^34^.

Taken together, the characteristics of the bedbug-*Wolbachia* system are more similar to those of nematodes than other arthropods (primary vs. secondary symbiosis, generic benefits vs. mutualism, complete vs. partial prevalence). Here we test whether these characteristics are reflected in the predicted co-speciation of bedbugs and *Wolbachia*. If so, it is possible that related bedbug species use a similar type of *Wolbachia* mutualism based on vitamin provision. We predict that a) a 100% prevalence of *Wolbachia* within and among bedbug species, b) co-speciation of *Wolbachia* and their bedbug hosts and c) that across bedbug species, the presence and potential function of the biotin genes shows evidence for a beneficial relationship.

## Material and Methods

### Samples

Sampling was restricted to the subfamily Cimicinae and covers all close relatives of the human-associated *C*. *lectularius*, a lineage with a known *Wolbachia* status^31,33^. The samples comprise the bat-associated lineage of *C*. *lectularius*, the sister species *C*. *emarginatus* (S. Roth *et al*., unpublished), and representatives of the three remaining species groups^35^: *C*. *pilosellus*, *C*. *pipistrelli*, *C*. *hemipterus*, as well as two bird-associated species: *C*. *hirundinis*, *C*. *vicarius* (formerly classified as *Oeciacus* – see^36^). Three species of the closely related genus *Paracimex* are included as is another bird related *Cimex* sp. from Japan (Table S1). Specimens were morphologically identified using Usinger’s^35^ and Ueshima’s^37^ keys and compared against an available phylogenetic data base (S. Roth *et al*., unpublished).

Individuals of each species were collected from as many locations as possible, up to ten, in order to obtain a reliable estimate of intraspecific genetic diversity and a meaningful estimate of prevalence of *Wolbachia* in bedbug populations. For *C*. *lectularius* whose *Wolbachia* infection status is already known, we analysed one human-associated population and three bat-associated populations. A list of all samples used in this study can be found in Table S1.

### DNA analyses

DNA was extracted from longitudinal halves of insect bodies in order to cover all insect tissues and account for all possible bacterial fauna. We extracted DNA for all samples using the DNeasy Blood & Tissue kit (Qiagen) and stored the DNA at −18 °C prior to use. To assess the infection status and reconstruct the phylogeny of the bedbug- specific *Wolbachia* strain, we studied two *Wolbachia* loci: the surface protein gene (WSP) and another protein-coding locus (HCPA). Both loci are widely used to characterize infection rate and describe phylogenetic relationships^38^. To track the beneficial relationship of *Wolbachia* to bedbug hosts, we chose two biotin loci: BioC and BioH. Each of these was previously found to contain a frameshift in *Wolbachia* of *C*. *japonicus* compared to the fully functional coding sequence in *C*. *lectularius*^33^, suggesting a loss of function in *C*. *japonicus*.

For WSP and HCPA loci, we used the universal primers designed for strain typing^38^. For each of the biotin loci, a pair of primers was designed to cover the whole coding region. For the bedbug phylogeny, we chose two mitochondrial loci, as mtDNA was shown to provide results congruent with a multilocus approach^36^ and exhibit the same inheritance mode as the *Wolbachia* infection. For species identification, we used the barcoding fragment of cytochrome oxidase subunit I (658 bp). For specimens chosen for the co-cladogenesis test, we used also a fragment of 16 S (386 bp). All primers used are listed in Table S2.

The target loci were amplified in 50 ul using GoTaq polymerase (Promega), recommended concentrations of other reagents and 2–4 ul of genomic DNA. The annealing temperature for each primer pair is given in Table S2. Purified PCR products were analyzed through a commercial sequencing service (Macrogen Inc. or GATC Biotech).

The HCPA sequences of samples 005 and 069 in *C*. *pipistrelli* and 120, 129 and 130 in *C*. *hirundinis* showed a secondary signal pointing to an infection by another *Wolbachia* strain (see Results). We validated by sequencing one sample per species using a newly designed primer HCPA-R2 (see Table S2) which specifically amplified only a single variant.

### Phylogenetic analyses

The alignment of the sequence data was carried out using MAFFT^39^. The phylogenetic analyses were run in IqTree^40^ and MrBayes 3.0^41^. Each analysis was carried out three times in order to assess convergence. The IqTree was used to infer Maximum Likelihood (ML) phylogenetic trees, using default settings, automatic model choice and 1000 bootstrap alignments. The Bayesian analyses were run using GTR (Generalised Time Reversible) model with gamma-distributed rate variation across sites and a proportion of invariable sites, both with default priors and priors set according to maximum model probability by sampling across GTR model space (lset nst = mixed rates = gamma). The MCMC chain was run in two simultaneous and independent runs for at least 2 million generations in order to achieve a standard deviation of split frequencies below 0.01. For the consensus tree, 10% of trees with unstable probability values were discarded as burn-in.

We chose 7 insect taxa with *Wolbachia* infections to serve as outgroup samples for phylogenetic analyses, according to a) similarity of the WPS sequence to *C*. *lectularius* associated *Wolbachia* and b) availability of HCPA sequence for the same sample.

The *Wolbachia* loci WSP and HCPA could have been affected by possible recombination between bacterial strains^38^. In order to reconstruct the *Wolbachia* phylogeny and test the co-cladogenesis with bedbug hosts, we used both loci as separate datasets as well as a concatenated dataset and compared the results. The two biotin loci are overlapping regions and were therefore analysed together as a partitioned dataset with independent model parameters for each gene. The two bedbug mitochondrial genes also do not recombine, therefore the same procedure was applied. Among the gene coding sequences, no gene alignment within the bedbugs using the primary sequence signal contained indels, however, the WSP alignment did when outgroups were included. From individuals that showed a secondary signals, we used only the sequence of the primary signal, after it was re-sequenced with the newly designed primer HCPA-R2 (see DNA analyses; Table S2).

In order to test for co-cladogenesis between *Wolbachia* and the host, we used the I_cong_ index^42^, a topology-based method, and TreeMap 3.0^43^ which encompasses both distance- and event-based algorithms. In addition, we used Tredist function in Phylip^44^ to assess the topological Robinson-Foulds distance^45^ of the mtDNA trees to the *Wolbachia* trees, comparing to distance to 1000 random trees generated by T-Rex^46^. For the *Wolbachia* trees, we used all unique combinations of WSP and HCPA sequences. The sequences of the secondary signal of the *C*. *pipistrelli* and *C*. *hirundinis* samples were not included (see Results). As the mtDNA variation within bedbug species was greater than that of *Wolbachia* sequences, we randomly assigned one of the corresponding mtDNA sequences to each unique *Wolbachia* sequence.

The included co-cladogenesis tests can use only binominal trees. Some of the Bayesian analysis produced polythomies at the terminal tree nodes; we therefore randomly deleted an appropriate number of taxa from the trees to be used. The final count of tree tips is given in Table 1. Using the I_cong_ index, we counted the number of tree tips in the Maximum Agreement SubTree (MAST), and calculated the probability that the *Wolbachia* and bedbug trees were congruent by chance. The Treemap was used to test the significance of the number of co-divergence events by the Patristic Distance Correlation Test and the significance of the tree congruence was determined by comparing 1000 random *Wolbachia* trees, taking into account the timing of both bedbug and *Wolbachia* phylogenies, Priors were set as recommended by the preliminary mapping analysis.

**Table 1:** Results of co-cladogenesis tests.

Dataset for tree construction	WSP		HCPA		Concatenated	
Phylogenetic method	Bayes	ML	Bayes	ML	Bayes	ML
No. of tree tips*	14	18	11	14	16	20
**I**_**cong**_ **results**						
I_cong_ index	1.842	1.783	1.667	1.474	1.893	1.536
No. of tree tips in The Maximum Agreement SubTree	10	11	8	8	11	10
Significance (p-value)	0.000020	0.000024	0.000311	0.002727	0.000007	0.000650
**Treemap results**						
Max. lineage codivergence events	19	19	15	19	27	24
No. of significant pairwise co-divergence events	9	10	6	7	8	11
Significance by randomizing (1000×) the parasite tree	0.0080	0.0300	0.0000	0.0000	0.0000	0.0000
**Rating of** ***Wolbachia*** **tree among 1000 random trees based on Robinson-Foulds distance to the bedbug mtDNA tree**	**1**	**1**	**1–2**	**1**	**1**	**1**

*Polytomies were collapsed, i.e. this number is higher by 1 than the number of co-divergence events in case of perfect co-speciation.

Samples identified as *Paracimex borneensis* and *P*. *setosus* appeared likely to be a single species based on sequence data. Since the taxonomy of the genus requires a thorough revision, we retained the identification based on morphology and geography, though the two samples are dealt with as a single species in Table 2.

**Table 2:** Gene variation for species.

Species	No. of locations (no. of specimens, if more than no. of locations)	mtDNA and *Wolbachia* variation			Biotin status		Synonymous and nonsynonymous variation in biotin				Deletions in biotin loci			
		no. of mtDNA haplotypes (mean p-distance in COI)	WSP variation (no. of variants)	HCPA variation (no. of variants)	BioC	BioC	BioC		BioH		91th bp of 2nd BioH variant	475th bp of 2nd BioC variant	between 549–555th bp of 2nd BioC variant	close to BioC forward primer
							nonsyn	syn	nonsyn	syn				
*Paracimex setosus/borneensis* ^*x*^	2	2 (0.000)	2	0	**eroded**	**distant** ^**xx**^	**eroded**		**0**.**0905**^**xx**^	**0**.**2714**^**xx**^	na	na	na	na
*Paracimex* cf. *chaeturus*	1 (2)	1	1	1	**distant**	**eroded**	**0**.**0400**	**0**.**1497**	**eroded**		na	?	?	?
*Cimex adjunctus*	9	8 (0.011)	2	2	functional	functional	0.0037	0.0050	0.0066	0.0060	na	na	na	na
*Cimex antennatus*	1	1	1	1	functional	functional	0.0037^xxx^	0.0000^xxx^	0.0044	0.0060	na	na	na	na
*Cimex brevis*	10	5 (0.004)	1	1	functional	functional	0.0056	0,0000	0.0044^xxx^	0.0308^xxx^	na	na	na	na
*Cimex latipennis*	2	2 (0.016)	1	1	functional	functional	0.0093	0.0151	0.0022	0.0246	na	na	na	na
*Cimex hemipterus*	7 (8)	2 (0.004)	2	1	**distant**	**distant**	**0**.**0404**	**0**.**0952**	**0**.**0296**	**0**.**0319**	all samples	all samples	no deletion	no deletion
*Cimex lectularius*	4	4 (0.013)	2	1	functional	functional	0.0037	0.0050	0.0022	0.0060	na	na	na	na
*Cimex emarginatus*	1	1	1	1	functional	functional	0.0037	0.0050	0.0044	0.0060	na	na	na	na
*Cimex pipistrelli*	13	10 (0.018)	2	2	functional	functional	0.0056	0.0050	0.0088	0,0000	all samples	all samples	no deletion	all samples
*Cimex japonicus*	2	1	1	1	functional	functional	0.0056	0.0050	0.0066	0,0000	all samples	all samples	no deletion	all samples
*Cimex hirundinis*	5	2 (0.003)	2	1	functional	functional	0.0056	0.0050	0.0088	0.0060	no deletion	all samples	all samples	no deletion
*Cimex vicarius*	2 (3)	3 (0.002)	2	1	functional	functional	?				at least 3 deletions at different positions			
*Cimex* sp. Japan	3 (4)	1	1	1	functional	functional	0.0056	0.0050	0.0066	0.0000	all samples	all samples	no deletion	no deletion

^x^The two *Paracimex* species delimited according to^37^ appeared to be a single species according to mtDNA and *Wolbachia* sequences.

^xx^based on 186 bp fragment amplified by BioC primers.

^xxx^value incl. *C*. *adjunctus* sample N1. The *Wolbachia* of the sample N1 clustered with *C*. *brevis* based on WSP and HCPA loci, and is identical to *C*. *brevis* in BioH sequence, but to *C*. *antennatus* in BioC sequence. In BioC *C*. *brevis* and *C*. *antennatus* differ only in a single nucleotide. Therefore the identity of BioC of sample N1 to *C*. *antennatus* may likely be a convergence.

eroded = contained deletions or stop codons, therefore it was not possible to use the same method for computing the syn and nonsyn mutations.

na = secondary signal not present.

bold = biotin loci concluded to be non-functional.

### Test of function of biotin loci

We used codon structure integrity of BioC and BioH sequences, and their relative divergence from a common ancestor, to infer the functionality of biotin production of *Wolbachia*. *Paracimex* cf. *chaeturus* and all samples within the clade consisting of *Cimex hemipterus*, C. *pipistrelli* group and all bird-related *Cimex* species provided sequences with two or more overlaying signals. These sequences however clearly represented the same variants differing in length, i.e. deletions were present in some. Most samples within species showed the full-length variant to be clearly dominant and the secondary signal to be of a consistent peak height. Therefore, we could easily separate the full-length sequence from the dominant signal as only two locations were available and the secondary signal was strong, except for in *C*. *vicarius*. As an additional safeguard, we manually reviewed the sequence by chromatogram inspection using CodonCode Aligner 6.0.2. The secondary signal was synchronous with the full-length variant until the deletion in the direction of reading. Since all samples were sequenced from both ends of each locus, for each part of the sequence a synchronous and asynchronous double signal was available. The synchronous signal served to detect the variable sites, as well as to correct for within-species variation. The asynchronous signal then served to assess the correct nucleotide at each of the variants. Only two sites in *C*. *pipistrelli*, three in *Cimex* sp. Japan and six in *C*. *japonicus* remained ambiguous in the full-length variants.

**Figure 1:**
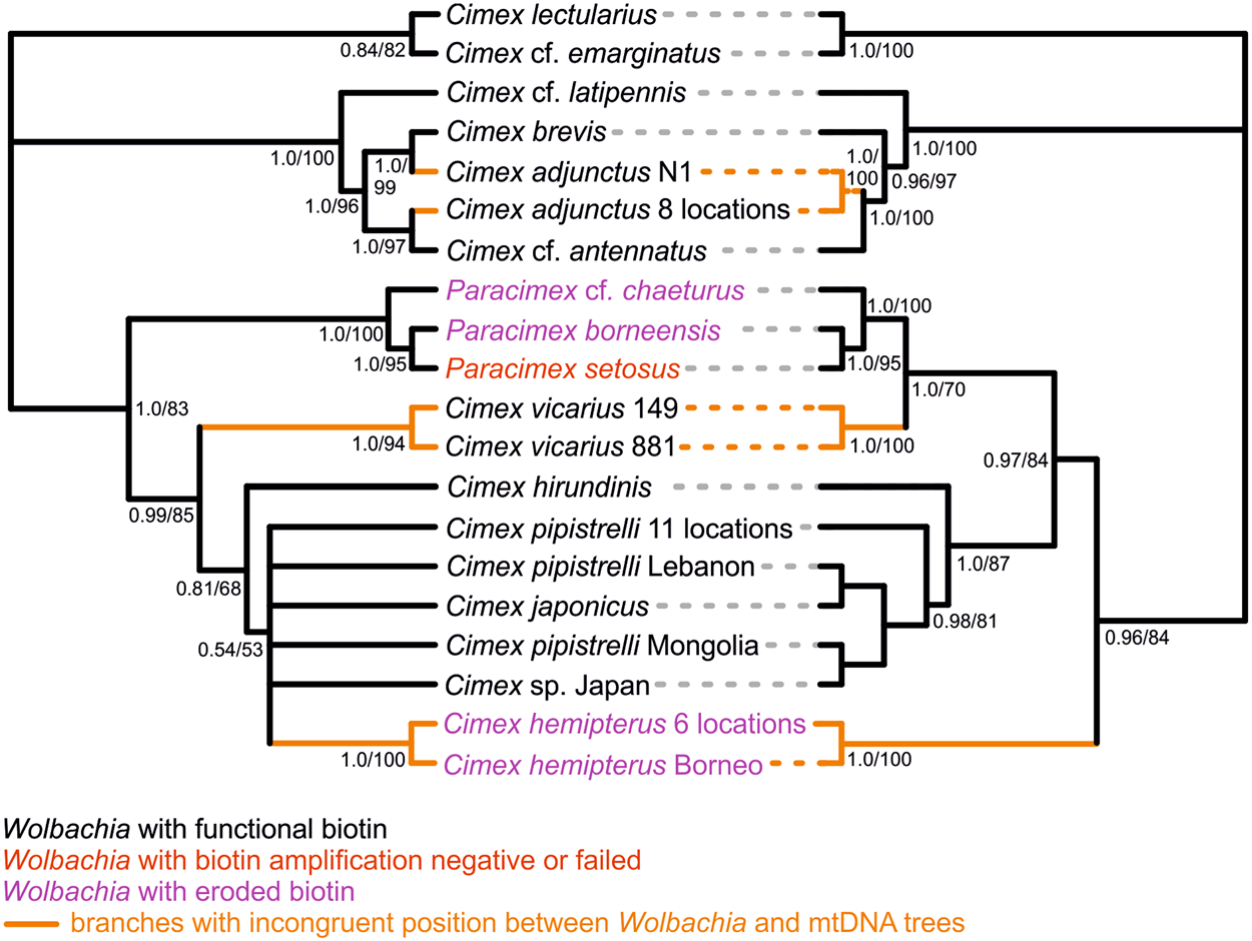
Cladograms based on mtDNA (right) and *Wolbachia* WSP and HCPA loci (left). Bayes posterior probability values are given at each node. For congruence test resuls see Table 1. All unique combinations of sequences of *Wolbachia* were used, along with mtDNA of corresponding samples. Labels after species name refer to collection site (Table S1). Highlighted are the different positions of a) *Cimex adjunctus* sample N1, b) *C*. *vicarius* and c) *C*. *hemipterus*.

In order to test whether *Wolbachia* biotin loci are functional and underlie the symbiotic relationship in bedbug species other than *C*. *lectularius*^31^, we compared all sequences with the sequence of a presumed common ancestor of the sampled species. We reconstructed the ancestral sequences for the biotin loci based on sequences that contained no frame shift or no stop codons, using Mega 7.0^47^. As the node representing the common ancestor we naturally chose the trichotomy between clades consisting of a) *C*. *pilosellus* group, b) *C*. *lectularius* group and c) a clade consisting of *C*. *pipistrelli* group, *C*. *hemipterus* and the bird-associated *Cimex* species (*Cimex* sp., *C*. *vicarius* and *C*. *hirundinis*). These three clades correspond to traditional systematics of the genus *Cimex* and consistently show monophylies in phylogenetic studies, but the relationships among them remain unresolved (Figs 1 and S1^36^, S. Roth *et al*. unpublished). The divergence in synonymous and non-synonymous changes was computed in PAML 4.8^48^ using the method by Yang & Nielsen^49^.

### Data availability

The DNA sequences of both bedbugs and *Wolbachia* used in the study are available at GenBank under codes provided in Table S1.

## Results

### Wolbachia prevalence

All 68 individuals of all 15 bedbug species tested were *Wolbachia*-positive (Figs 1 and S1, Table 2). Except for five samples (2/13 from *C*. *pipistrelli*, 3/5 from *C*. *hirundinis;* see next paragraph), both WSP and HCPA genes showed unambiguous sequences of a single *Wolbachia* strain present. In those five samples, only HCPA showed a double signal, According to the consistence of the peak heights, this double signal suggests the presence of two *Wolbachia* strains. The dominant signal belonged to the same *Wolbachia* strain that was found in other bedbug species. The variation of WSP and HCPA loci of this strain within bedbug species was either zero or very low (Table 2). The low diversity of WSP among the bedbug species allowed for visual inspection of alignment and let us conclude that no codon mismatch or recombination across the WSP sequence had occurred.

The HCPA sequences reconstructed from the secondary signal of the five samples of *C*. *hirundinis* and *C*. *pipistrelli* pointed to a co-infection by another strain. The sequence and the relative strength of the signal was consistent across specimens of each species, all coming from different geographic locations. It always represented *Wolbachia* infections belonging to supergroups other than supergroup F according to a clustering with the outgroups (Fig. S1; for sample codes see Table S1). We therefore have solid reasons to believe that the secondary signal represented a single less abundant *Wolbachia* strain in each *Cimex* species. *Wolbachia* supergroups other than F have not been previously found to provide benefits to bedbugs and were, therefore, not tested for co-phylogeny with bedbugs.

### Co-cladogenesis

Each dataset (*Wolbachia* WSP, HCPA, WSP + HCPA, *Wolbachia* biotin loci, bedbug mtDNA) produced the same topology and very similar posterior probability or bootstrap values across across all runs and priors, either in Bayesian or ML analyses (Figs 1, S1 and S2). The trees based on a particular dataset produced by Bayesian analyses were sometimes less resolved than the ML ones, but otherwise no conflicts in topology were observed. The only topology differences between trees based on different *Wolbachia* datasets were the varying positions of *Cimex hemipterus* and *C*. *vicarius*.

The bedbug and *Wolbachia* trees were clearly congruent (Fig. 1). Both Treemap and I_cong_ co-cladogenesis tests unambiguously supported a close congruence of bedbug and *Wolbachia* phylogenies (Table 1). Among 1000 random trees, the Robinson-Foulds distance of the *Wolbachia* to the mtDNA trees was always lowest using either Bayesian or ML trees datasets based on any of the *Wolbachia* datasets.

Across all the six of bedbug mtDNA tree with the *Wolbachia* trees comparisons (three *Wolbachia* datasets, two phylogenetic methods), positions of only three taxa differed. Two of them, however, represented unstable topologies across different *Wolbachia* trees as well (see Discussion).

### Biotin function

The *Wolbachia* biotin loci were successfully amplified in all but two specimens. In one *P*. *borneensis* specimen (C94) the BioH primers failed and in *P*. *setosus* (C9) neither locus was amplified despite repeated trials. It is not possible to determine whether the failure to amplify was caused by poor DNA quality, the absence of the biotin loci or primer mismatch due to sequence divergence. However, amplification of the HCPA locus using general primers failed as well in these two specimens.

*Wolbachia* in all samples within the clade consisting of *Cimex hemipterus*, C. *pipistrelli* group and the bird-related *Cimex* species showed at least one additional variant of the biotin loci. The secondary signal was present in *Paracimex* cf. *chaeturus* but only visible when the BioC reverse primer was used for sequencing. These variants contained deletions and the pattern of the distribution of the deletions along the genes was largely consistent across species (Table 2). The two deletions in biotin genes found in *C*. *japonicus* in a previous study^33^ were found in the secondary signal of biotin in our samples as well, along with two other deletions.

Biotin sequences drawn from the dominant signal showed low divergence from the presumed ancestral sequence and were similar among most of the bedbug species (Table 2, Fig. S2). No changes in codon structure were detected. In all *Paracimex* species and in *C*. *hemipterus*, BioC sequences contained frameshifts in all variants detected. The divergence from the common ancestor of the sequences drawn from the dominant signal was one order of magnitude higher than in the rest of the species.

## Discussion

Our study revealed two patterns of *Wolbachia* infection in bedbugs that proved to be unique to arthropods and that have previously been thought to be typical for, and restricted to, nematodes. First, as predicted, we showed a 100% prevalence of *Wolbachia* within and among the sampled bedbug species. This contrasts with reports from other arthropods showing infection rates that are typically either below 10%, or above 90% within species^19,20,50,51^, although infections up to 100% have been observed even within presumably parasitic relationships^52,53^. A high prevalence is predicted in *Wolbachia*-nematode relationships, where *Wolbachia* are usually present in all individuals^16,17^ and where all genera or larger monophyletic clades are infected^9^.

Secondly, we show, by various methods, the predicted very close co-cladogenesis between bedbugs and *Wolbachia*, which is a unique finding among arthropods, but is the norm in filarial nematodes and their *Wolbachia* symbionts^9,18^. One potential case of co-speciation has been reported in arthropods, namely that between *Wolbachia* and three *Nasonia* wasp species^27^ although horizontal transfer of *Wolbachia* was not ruled out. Our results show significant congruence between *Wolbachia* and the bedbug phylogenies in 15 species, regardless of whether any of the *Wolbachia* loci or the concatenated dataset were used. Three cases of incongruence between bedbug and *Wolbachia* phylogenies were observed in each analysis (Fig. 1). While such incongruence may be attributable to limited molecular information used in our study, all three cases are also consistent with alternative hypotheses. In one case, the position of *Cimex vicarius* varied between the *Wolbachia* and bedbug phylogenies, as well as among different *Wolbachia* trees. The position of *C*. *vicarius* has been very unstable even in previous multilocus phylogeny reconstructions^36^, possibly due to a conflict between nuclear and mitochondrial molecular data. *Wolbachia* molecular information is therefore not fully comparable. In the second case, the position of *C*. *hemipterus* varied among *Wolbachia* trees as well. Non-functional biotin provision by *Wolbachia* and consequently less stringent symbiosis with a host and a different evolutionary divergence are attributable in this case. The third case likely represents a transfer of *Wolbachia* from *C*. *brevis* to the population of *C*. *adjunctus* (sample N1, Fig. 1), a possibility facilitated by the fact that the two species are phylogenetically closely related^36^ and live in geographic vicinity^35^.

Both results, the complete prevalence and the co-cladogenesis with the host, are predicted^4^ if, as has previously been found, the bedbug-*Wolbachia* relationship is based on mutualistic nutritional provision^31^. The fact that both predictions were upheld simultaneously may indicate that both phenomena are related and may be part of a syndrome characterising the transition from detrimental to beneficial *Wolbachia*. Two other characters are also correlated with the type of symbiosis and we propose they are part of a syndrome. Nematode-*Wolbachia* relationships are strictly vertically inherited, whereas horizontal transmission between arthropod species or populations are common^5^. Finally, nematode individuals or species are infected by single strains, while infections by multiple strains are common in arthropod species. However, despite the striking parallel between the bedbug and the nematode-associated obligate nutrient provision, co-cladogenesis was not entirely congruent between bedbugs and *Wolbachia*. Five samples, corresponding to two species (*C*. *hirundinis* and *C*. *pipistrelli)* showed evidence of an infection with two phylogenetically distant *Wolbachia* strains (Fig. S1). It is currently not clear whether both strains are involved in nutrition provision or represent a case of competition between two strains that would be resolved over time (with the predicted outcome of the mutualistic strain outcompeting the non-mutualistic one^4^;). The occurrence of two strains also suggests that horizontal transfer of *Wolbachia* between bedbug species may not be impossible although it is not currently clear how a horizontal transfer might have happened between *C*. *adjunctus* and *C*. *brevis* (Fig. 1). Sterile interspecific matings have been observed in several bedbug species, including between *C*. *adjunctus* and *C*. *brevis*^35^, though sexual transmission of *Wolbachia* has to our knowledge not previously been observed.

It may also be important to note that while strict co-speciation is considered ubiquitous for *Wolbachia*-nematode associations^14,18^, the supergroup F, to which the bedbug-associated *Wolbachia* belongs^31^, shows considerable breakdowns of co-speciation patterns with the nematode hosts^17,54^. In such context, the co-cladogenesis of *Wolbachia* with bedbugs that we showed may represent a case of the tightest co-phylogeny recorded for the *Wolbachia* supergroup F.

We found no evidence of an erosion of the protein coding structure in the *Wolbachia* biotin loci, including in the *Wolbachia* of *C*. *japonicus*. Dissimilar to the study of Nikoh *et al*.^33^, we conclude that the *Wolbachia* biotin synthesis is still in function in most *Cimex* species (except *C*. *hemipterus*, see below). The difference between the two studies may have arisen because of intraspecific variation of *Wolbachia* in *C*. *japonicus* or, alternatively, because our study may have used primers with different specificity than Nikoh *et al*.^33^ had.

We observed additional variants of the biotin loci in a monophylum consisting of all bird-associated species, the *C*. *hemipterus* and *C*. *pipistrelli* species group (i.e. including *C*. *japonicus*, see Table 2). These biotin variants contained frameshifts similar to those found in *C*. *japonicus* by Nikoh *et al*.^33^ and did not represent functional genes. Since functional variants were also present in most of these hosts, the presence of the deleterious ones does not suggest an arrest in biotin provision by *Wolbachia* to the bedbugs. The deleterious variants are unlikely to originate from co-infecting *Wolbachia* strains, because WSP and HCPA loci showed no evidence of a secondary infection in the respective specimens, except in the previously mentioned samples of *C*. *hirundinis and C*. *pipistrelli*. However, it is noteworthy that the positions of the deletions in the biotin sequence was very similar among species (Table 2), strongly suggesting a common origin of the deleterious variants within the species clade, though the actual location of the variants remains unknown.

We found deleterious and non-functional sequences exclusively in *Cimex hemipterus* and in two *Paracimex* species. The difference between these two groups and the rest of *Cimex* is further supported by the length of branches of these taxa on the HCPA and WSP trees (Fig. S1), compared to the length of branches in other *Cimex* species with functional biotin. While most bedbug species in our analysis use biotin provision by *Wolbachia*, the *Wolbachia* symbiosis in *C*. *hemipterus* and *Paracimex* spp. is likely evolutionarily different, perhaps dependent on other resources provisioned by *Wolbachia*, such as riboflavin. This idea may be tested by experimental using *Wolbachia* manipulations in *C*. *hemipterus* and *Paracimex*.

## Conclusions

Regardless of the discussed details, our data provide clear evidence that the syndrome of transition from a host-detrimental to a host-beneficial relationship has evolved in convergence in both bedbugs and filarial nematodes. Both bedbugs and filarial nematode show 100% *Wolbachia* prevalence and strict co-speciation of *Wolbachia* and the host taxa. This is exceptional among arthropods, and bedbugs therefore offer a valuable example of evolution of symbiotic relationships. Given the recently proposed possibilities that nematodes that are harmful to humans may be controlled with antibiotics targeting their *Wolbachia*, it would be interesting to explore whether such a possibility exists in bedbugs, too.

## Electronic supplementary material

Supplementary information
