# Supplementary material for: Co-speciation in bedbug Wolbachia parallel the pattern in nematode hosts
Source: Sci Rep. 2018 Jun 11;8:8797. doi: 10.1038/s41598-018-25545-y (PMC5995804; doi:10.1038/s41598-018-25545-y)
Supplement: Supplementary file 1 — Supplementary information [file 41598_2018_25545_MOESM1_ESM.pdf]

Supplementary information to the manuscript titled:

Co-speciation in bedbug *Wolbachia* parallel the pattern in nematode hosts

Authors: Ondřej Balvín, Steffen Roth, Benoit Talbot, Klaus Reinhardt

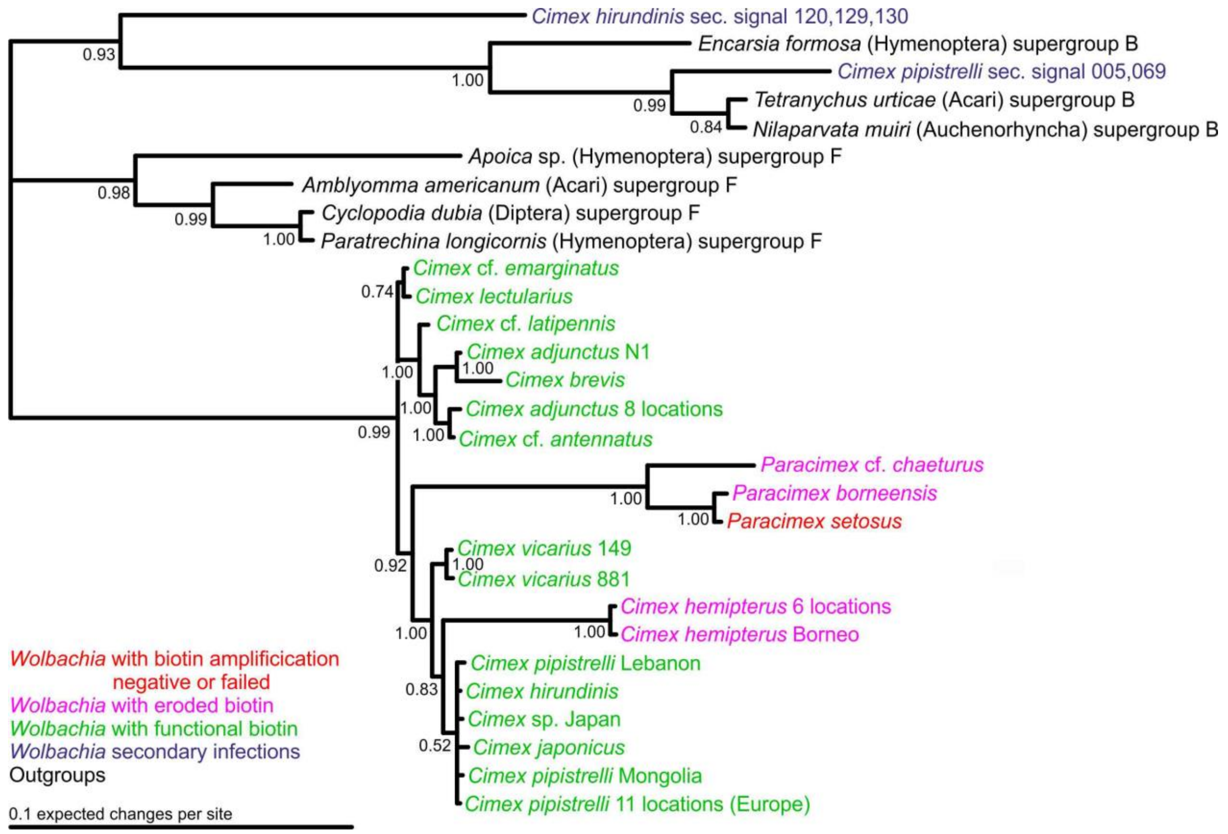

Fig. S1. *Wolbachia* phylogenetic tree based on concatenated HCPA and WSP datasets. Only unique combinations of sequences were used. Labels after bedbug species name refer to collection site (Table S1).

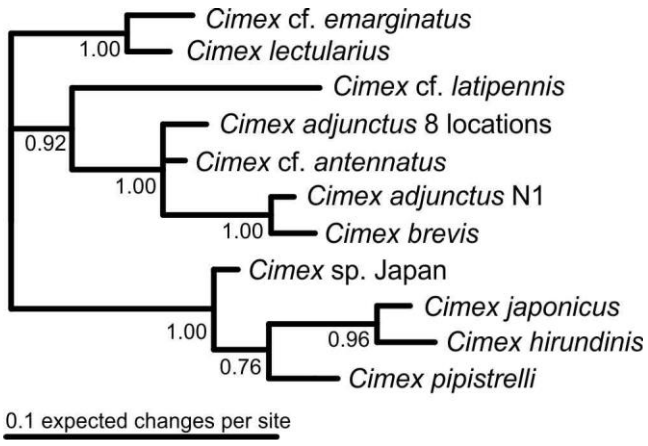

Fig. S2. A tree based on sequences of the BioC and BioH loci.

						Genbank accession numbers					
**Sample code**	**Country**	**Locality**	**Date**	**Host**	**Collector**	**Bedbug COI**	**Bedbug 16S**	**Wolbachia hcpA**	**Wolbachia hcpA, secondary infection**	**Wolbachia WSP**	**Wolbachia biotin**
**Cimicinae: *Cimex adjunctus* Barber, 1939**											
140	USA	Washington county, North Carolina	07.06.2005	*Nycticeius humeralis*	Matina Kalcounis-Ruppell	GU985536	GU985558	MF662572		MF687874	MF687889
141	USA	Galesburg, Kalamazoo county, Michigan	06.07.2005	*Eptesicus fuscus*	Lee Johnson	GU985535	GU985557	MF662572		MF687874	MF687889
142	USA	Fulton, Kalamazoo county, Michigan	19.06.2005	*Eptesicus fuscus*	Lee Johnson	GU985537	GU985559	MF662572		MF687874	MF687889
N1	Canada	Northwest Territories	16.06.2014	*Myotis lucifugus*	Laura Kaupas	KU534908		MF662571		MF687873	MF687890
RH01	USA	Attica, Lapeer county, Michigan	27.07.2018	*Eptesicus fuscus*	Maarten Vonhof	KU534912		MF662572		MF687874	MF687889
RG08	USA	Larimer county, Colorado	2005	*Eptesicus fuscus*	Roger Pearce, Maarten Vonhof	KU534932		MF662572		MF687874	MF687889
EFWN		Prairie Creek, Vigo county, Indiana	13.07.2005	*Eptesicus fuscus*	Lee Johnson, John Whitaker, Maarten Vonhof	KU534927		MF662572		MF687874	MF687889
MV642	USA	Bourbon county, Kentucky	17.08.2010	*Eptesicus fuscus*	Elizabeth Warburton, Maarten Vonhof	KU534914		MF662572		MF687874	MF687889
CCC		Canoe Creek State Park, Blair county, Pennsylvania	30.05.2006	*Myotis lucifugus*	Cal Butchkoscki, Maarten Vonhof	KU534908		MF662572		MF687874	MF687889
**Cimicinae: *Cimex* cf. *antennatus* Usinger and Ueshima, 1965**											
C10	USA	Antelope Valley, California	15.08.2002	unknown bat host	A.C. Lohmann	KF018760	KF018732	MF662573		MF687875	MF687891
**Cimicinae: *Cimex brevis* Usinger and Ueshima, 1965**											
N2	Canada	Northwest Territories	20.06.2014	*Myotis lucifugus*	Laura Kaupas	MF680523		MF662574		MF687876	MF687892
5571	Canada	Londonderry, Colchester, Nova Scotia	21.08.2009	*Myotis septentrionalis*	Hugh Broders	MF680522		MF662574		MF687876	MF687892
11540	Canada	Cache River, Labrador	12.07.2012	*Myotis lucifugus*	Lynne Burns, Tony Parr, Hugh Broders	MF680521		MF662574		MF687876	MF687892
7196	Canada	Wabush, Lac Joseph, Labrador	25.06.2011	*Myotis lucifugus*	Sara McCarthy, Hugh Broders	MF680521		MF662574		MF687876	MF687892
C20	Canada	Montana		*Eptesicus fuscus*	C. Lausen	KF018759	KF018731	MF662574		MF687876	MF687892
O9	Canada	Bruce Peninsula National Park, Ontario	08.10.2014	*Lasionycteris noctivagans*	Kristin Jonasson	KY561679		MF662574		MF687876	MF687892
1721	Canada	Dollar Lake Provincial Park, Halifax, Nova Scotia	20.06.2006	*Myotis septentrionalis*	Hugh Broders	MF680523		MF662574		MF687876	MF687892
7513	Canada	Londonderry, Colchester, Nova Scotia	19.08.2010	*Myotis septentrionalis*	Hugh Broders	MF680522		MF662574		MF687876	MF687892
7909	Canada	Dutch Settlement, Halifax, Nova Scotia	09.09.2010	*Myotis septentrionalis*	Hugh Broders	MF680522		MF662574		MF687876	MF687892
11506	Canada	Minipi, Labrador	09.07.2012	*Myotis lucifugus*	Lynne Burns, Tony Parr, Hugh Broders	MF680521		MF662574		MF687876	MF687892
**Cimicinae: *Cimex* cf. *emarginatus* Simov, Ivanova & Schunger, 2006**											
431	Maroque	Bouhachen, Rif	06.05.2013	*Pipistrellus pipistrellus*	Tomáš Bartonička	MF680526	MF680517	MF662576		MF687880	MF687893
**Cimicinae: *Cimex hemipterus* Fabricius, 1803**											
092	India	Mannárakkát	29.09.2005	humans	Petr Šípek	GU985538	GU985560	MF662585		MF687878	MF687900
093	Indonesia	Kuala Lumpur	2006	humans	Magdalena Lučanová	KF018754	GU985560	MF662585		MF687878	MF687900
148	Indonesia	Jakarta, Java, hotel	2008-2011	humans	Petr Šrámek	KF018754		MF662585		MF687878	MF687900
348	Malaysia	Kotakinabalu, Borneo, hotel	May 2011	humans	Petr Šrámek	KF018754		MF662585		MF687878	MF687900
349	South East Asia	unknown locality	2011	humans	Petr Šrámek	KF018754		MF662585		MF687878	MF687900
801	collection information missing			humans	Robert Vlk	KF018754		MF662585		MF687878	MF687900
347	Malaysia	Kota Kinabalu, Sabah, Borneo, hotel	summer 2015	humans	Martina Komárková	KF018754		MF662585		MF687879	MF687900
**Cimicinae: *Cimex* (former *Oeciacus*) *hirundinis* Lamarck, 1816**											
120	Czech Republic	Žíteč, Jindřichův Hradec district	23.11.2007	*Delichon urbica*	Jaroslav Cepák	GU985543	GU985565	MF662580	MF662578	MF687887	MF687896
129	Germany	Postdam - Golm	17.8.2007	*Delichon urbica*	Ingo Scheffler	GU985544	GU985565	MF662580	MF662578	MF687887	MF687896
130	Germany	Postdam, Neu Fahrland	20.10.2007	*Delichon urbica*	Ingo Scheffler	GU985544		MF662580	MF662578	MF687887	MF687896
893	Czech Republic	Čížov, Znojmo district	26.5.2010	*Delichon urbica*	Antonín Reiter	GU985544		MF662580		MF687887	MF687896
890	Czech Republic	Karlovy Vary	summer 2015	*Delichon urbica*	Mrs. Vyklická	GU985544		MF662580		MF687887	MF687896
**Cimicinae: *Cimex japonicus* Usinger, 1966**											
350	Japan	Akita prefecture, Daisen city, Omagari bridge, bat roost	10.5.2010	*Vespertilio superans*	Mitsuru Mukohyama	KC503541	KF018727	MF662582		MF687883	MF687894
351	Japan	Aomori prefecture, Shichinohe town, Temmadate bathouse	3.8.2010	*Vespertilio superans*	Mitsuru Mukohyama & Tomoya Kobayashi	KC503541	KF018727	MF662582		MF687883	MF687894
**Cimicinae: *Cimex* cf. *latipennis* Usinger and Ueshima 1965**											
c18	Canada	British Columbia		unknown bat host	T. Luszczell	KF018758	KF018734	MF662575		MF687877	MF687895
c19	Canada	British Columbia		*Myotis volans*	T. Luszczell	KF018757	KF018733	MF662575		MF687877	MF687895
**Cimicinae: *Cimex lectularius* Linnaeus 1758**											
089	Iran	Golestan province, picnic grass field	27.05.2006	unknown host, bats according to morphology	Jiří Hájek, Pavel Chvojka	MF680527	MF680518	MF662577		MF687880	AP013028
552	Czech Republic	Rokycany	2014	humans	Antonín Drozda	MF680529		MF662577		MF687880	AP013028
PF2	Czech Republic	Doubravník, Brno - country district, papermill	2014	*Myotis emarginatus*	Tomáš Bartonička	GU985526		MF662577		MF687880	AP013028
HF3	Czech Republic	Hanušovice, Šumperk district, church	2014	*Myotis myotis*	Tomáš Bartonička	MF680528		MF662577		MF687880	AP013028
**Cimicinae: *Cimex pipistrelli* Jenyns, 1839, event. C.pip group**											
005	Czech Republic	Točník, castle stairs	16.7.2005	*Myotis myotis*	Ondřej Balvín	GU985527	GU985549	MF662580	MF662581	MF687881	MF687897
008	Czech Republic	Beroun, elementary school attic	16.5.2005	*Columba livia*	Ondřej Balvín	GU985527	GU985549	MF662580		MF687881	MF687897
013	Czech Republic	Lužnice, Jindřichův Hradec district, Inst. of Botany of ASCR	28.5.2005	*Myotis naterreri*	Ondřej Balvín	KC503516		MF662580		MF687881	MF687897
021	Czech Republic	Úštěk, Litoměřice district, church attic	6.6.2005	*Myotis myotis*	Borek Franěk	KC503519		MF662580		MF687881	MF687897
049	Czech Republic	Bohdalice-Pavlovice, Vyškov district, post office attic	27.6.2006	*Myotis myotis*	Martin Pokorný, Ondřej Balvín	GU985529		MF662580		MF687881	MF687897
102	Bulgaria	Plovdiv, bridge	3.11.2005	*Nyctalus noctula*	E. Tilova	KC503516		MF662580		MF687881	MF687897
115	Czech Republic	Líšná, Přerov district, mist-netted bat	6.8.2007	*Nyctalus noctula*	Radek Lučan	KC503523		MF662580		MF687881	MF687897
138	Hungary	Eger, mist-netted bat	1.4.2006	*Nyctalus noctula*	Péter Estók	KC503530		MF662580		MF687881	MF687897
139	Hungary	Bánhorváti, Borsod-Abaúj-Zemplén district, church attic	13.7.2008	mixed bat colony (*Rhinolophus ferrumequinum, Rh. euryale, Myotis myotis, M. emarginatus*)	Péter Estók	KC503518		MF662580		MF687881	MF687897
153	Hungary	Baja, Bács-Kiskun district, Cserta-Duna áttöltés, mist-netted bat	21.7.2007	*Nyctalus noctula*	Tamás Görföl	KC503512		MF662580		MF687881	MF687897
054	Lebanon	Nahr es Safa, mist-netted bat	26.4.2006	*Nyctalus noctula lebanoticus*	Ivan Horáček	KC503517		MF662580		MF687881	MF687897
069	Slovakia	Nitra, park, mist-netted bat	21.8.2006	*Nyctalus noctula*	Martin Ševčík	GU985527	GU985549	MF662579	MF662581	MF687881	MF687897
onon	Mongolia	Onon Gol	1.6.2008	unknown bat host	D. Dolch	MF680530		MF662580		MF687882	MF687897
**Cimicinae: Cimex sp.**											
895	Japan	Kamionohara, Konda, Sasayama city, Hyogo pref., bird nest	9.3.2008	*Delichon dasypus*	Nobuhiko Kataoka	GU985542	GU985564	MF662582		MF687884	MF687888
896	Japan	Kamiichi, Niimi city, Okhama pref., bird nest	18.8.2008	*Delichon dasypus*	Nobuhiko Kataoka	GU985542	GU985564	MF662582		MF687884	MF687888
897	Japan	Hokodate, Kotaki, Kisakata, Nikaho city, Akita pref., bird nest	31.8.2007	*Delichon dasypus*	Nobuhiko Kataoka	GU985542	GU985564	MF662582		MF687884	MF687888
899	Japan	Kamionohara, Konda, Sasayama city, Hyogo pref., bird nest	3.7.2007	*Delichon dasypus*	Nobuhiko Kataoka	GU985542	GU985564	MF662582		MF687884	MF687888
**Cimicinae: *Cimex* (former *Oeciacus*) *vicarius* (Horvath 1890)**											
149	USA	Sarben, Keith County, Nebraska	2.10.2008	*Petrochelidon pyrrhonota*	Charles Brown	GU985541	GU985563	MF662584		MF687885	
881	USA	Keystone, Keith County, Nebraska	1.10.2008	*Petrochelidon pyrrhonota*	Charles Brown	specimen 881n3: KF018752; 881n1: KF018753	KF018722	MF662584		MF687886	
**Cimicinae: *Paracimex borneensis* Usinger 1959**											
C94	Malaysia	Borneo, Niah cave	26.06.2015	*Aerodramus salanganus*	Steffen Roth & A. Scheidt	KF018761	MF680519			MF687870	MF687898
**Cimicinae: *Paracimex setosus* Ferris and Usinger 1957**											
C9	collection information missing					KF018761	KF018735			MF687871	
**Cimicinae: *Paracimex* cf. *chaeturus* Ueshima 1968**											
Jian	China	Yunnan, Jiangshui, cave	May 2015	*Aerodramus brevirostris*	Ondřej Balvín	MF680531	MF680520	MF662570		MF687872	MF687899

Table S1. List of material used.

Reference for host assignment of sample 089 (*Cimex lectularius*): Balvín O, Munclinger P, Kratochvíl L, Vilímova J. 2012 Mitochondrial DNA and morphology show independent evolutionary histories of bedbug *Cimex lectularius* (Heteroptera: Cimicidae) on bats and humans. Parasitol Res. 111(1):457–69.

Name	Sequence 5' - 3'	Annealing temperature	Reference
Wolbachia surface protein			
wsp F1	GTCCAATARSTGATGARGAAAC	58°C	1
wsp R1	CYGCACCAAYAGYRCTRTAAA	58°C	1
Wolbachia HCPA gene			
Hcpa F1	GAAATARCAGTTGCTGCAAA	55°C	1
Hcpa R1	GAAAGTYRAGCAAGYTCTG	55°C	1
Hcpa R2	GTTCAATTCCTGTGGCGAAAA	55°C	Newly designed
Wolbachia biotin loci			
BioH F3348	TGTAGCCTGTACCTGTACCCA	56°C	Newly designed
BioH R4123	GTGTTTTGTCACGGTTGGGG	56°C	Newly designed
BioC F2742	CCAACGTCGGTATCTGTTCCT	56°C	Newly designed
BioC R3700	CAGCTACCACACATACCACT	56°C	Newly designed
Cimicidae cytochrome oxidase subunit I			
LepF	ATTCAACCAATCATAAAGATATNGG	42°C	2, modified
LepR	TAWACTTCWGGRTGTCCRAARAATCA	42°C	2, modified
Cimicidae 16S ribosomal gene			
16S LR-J	TTACGCTGTTATCCCTAA	48°C	3
16S LR-N	CGCCTGTTTATCAAAAACAT	48°C	4
Cimicidae 18S ribosomal gene			
18S-1	CTGGTTGATCCTGCCAGTAGT	48°C	5
18S-3	GGTTAGAACTAGGGCGGTATCT	48°C	5

Table S2. An overview of primers used and designed in the present study.

References:

1. Baldo L et al. 2006 Multilocus Sequence Typing System for the Endosymbiont Wolbachia pipientis. Appl. Environ. Microbiol. 72, 7098–7110. (doi:10.1128/AEM.00731-06)
2. Hajibabaei M, Janzen DH, Burns JM, Hallwachs W, Hebert PDN. 2006 DNA barcodes distinguish species of tropical Lepidoptera. PNAS 103, 968–971.
3. Kambhampati S, Smith PT. 1995 PCR primers for the amplification of four insect mitochondrial gene fragments. Insect Mol. Biol. 4, 223–236.
4. Simon C, Frati F, Beckenbach A, Crespi B, Liu H, Flook P. 1994 Evolution, weighting and phylogenetic utility of mitochondrial gene sequences and a compilation of conserved polymerase chain reaction primers. Ann. Entomol. Soc. Am. 87, 651–701.
5. Tian Y, Zhu W, Li M, Xie Q, Bu W. 2008 Influence of data conflict and molecular phylogeny of major clades in Cimicomorphan true bugs (Insecta: Hemiptera: Heteroptera). Mol. Phylogenet. Evol. 47, 581–597.
